# Utilizing Multimodal Logic Fusion to Identify the Types of Food Waste Sources

**DOI:** 10.3390/s26030851

**Published:** 2026-01-28

**Authors:** Dong-Ming Gao, Jia-Qi Song, Zong-Qiang Fu, Zhi Liu, Gang Li

**Affiliations:** School of Computing and Artificial Intelligence, Beijing Technology and Business University, No. 11 Fu Cheng Road, Haidian District, Beijing 100048, China; gaodonming@btbu.edu.cn (D.-M.G.); songjiaqi11234@163.com (J.-Q.S.); liuzhi2874@163.com (Z.L.); ligang@btbu.edu.cn (G.L.)

**Keywords:** canteen waste identification, sensor fusion, image, machine learning, multi-modal fusion

## Abstract

**Highlights:**

**What are the main findings?**

- The MobileNetV3 + EMA vision model achieved a peak accuracy of 99.46% under ideal lighting (120–240 cd m^−2^), while the multimodal logic fusion strategy improved classification accuracy by 39.5% in low-light conditions (12 cd m^−2^).
- Audio recognition using Fast Fourier Transform (FFT) and Support Vector Machine (SVM) maintained a stable accuracy of 0.80, serving as a reliable fallback when illumination conditions caused visual recognition to fail.

**What are the implications of the main findings?**

- The proposed environment-aware logic fusion framework effectively solves the problem of visual model failure caused by significant lighting fluctuations in 24/7 industrial food waste processing.
- Accurate real-time identification of waste textures enables the automated adjustment of equipment operating parameters, distinguishing between kitchen waste (requiring high pressure) and leftovers (requiring low pressure).

**Abstract:**

It is a challenge to identify food waste sources in all-weather industrial environments, as variable lighting conditions can compromise the effectiveness of visual recognition models. This study proposes and validates a robust, interpretable, and adaptive multimodal logic fusion method in which sensor dominance is dynamically assigned based on real-time illuminance intensity. The method comprises two foundational components: (1) a lightweight MobileNetV3 + EMA model for image recognition; and (2) an audio model employing Fast Fourier Transform (FFT) for feature extraction and Support Vector Machine (SVM) for classification. The key contribution of this system lies in its environment-aware conditional logic. The image model MobileNetV3 + EMA achieves an accuracy of 99.46% within the optimal brightness range (120–240 cd m^−2^), significantly outperforming the audio model. However, its performance degrades significantly outside the optimal range, while the audio model maintains an illumination-independent accuracy of 0.80, a recall of 0.78, and an F1 score of 0.80. When light intensity falls below the threshold of 84 cd m^−2^, the audio recognition results take precedence. This strategy ensures robust classification accuracy under variable environmental conditions, preventing model failure. Validated on an independent test set, the fusion method achieves an overall accuracy of 90.25%, providing an interpretable and resilient solution for real-world industrial deployment.

## 1. Introduction

Large-scale commercial food establishments and canteens generate significant amounts of waste, particularly in densely populated areas such as China. On-site reduction, pretreatment, or complete processing of food waste as a low-cost solution has increasingly gained attention and is being adopted by many catering projects. During the treatment process, different types of food waste require significantly different equipment operating parameters. Therefore, an identification system is necessary to determine the characteristics of food waste and adjust the treatment system’s operating parameters in real time. Food waste can be categorized into two types: kitchen waste generated in the kitchen and leftovers produced after meals [1,2]. For example, kitchen waste typically has a high moisture content, high fibre content, and large particle sizes, necessitating processing under high pressure [3]. In contrast, leftovers tend to have high viscosity, low fibre content, and a semisolid state, requiring processing under low pressure [4,5,6].

In the field of image-based waste sorting, Lecun et al. proposed the LeNet network, a classic structure of convolutional neural networks (CNNs) [7]. Krizhevsky et al. introduced AlexNet, which established convolutional neural networks as the core algorithmic model for image classification [8]. Simonyan et al. proposed the VGG network, which ushered in the era of stacked 3 × 3 convolutions [9]. He et al. developed the ResNet network to address degradation issues [10]. Deep learning technology has been widely applied in the field of waste recognition. International researchers, such as Yang et al., have captured images of recycled waste and categorized it into six types: glass, paper, metal, plastic, cardboard, and general waste. In a study on the texture classification of kitchen waste based on image and audio data via machine learning, Masand et al. proposed a deep learning model based on the EfficientNet architecture, which achieved 98% accuracy on the TrashNet dataset [11]. Kim et al. classified radioactive waste into eight categories, achieving a 99.67% accuracy rate via deep neural network image recognition [12]. Majchrowska et al. identified household waste and used EfficientNet-B2 to classify detected waste into seven categories, achieving a classification accuracy rate of 75% [13]. Hossen et al. proposed RWC-Net for classifying six different types of waste, achieving an overall accuracy rate of 95.01% [14]. Alrayes et al. employed a Vision transformer for automatic waste classification, achieving a classification accuracy rate of 95.8% [15]. Adedeji et al. utilized a 50-layer residual network (ResNet-50) for pretraining and tested it on the waste image dataset developed by Gary Thung and Mindy Yang, achieving an accuracy rate of 87% on the dataset [16]. In summary, while image recognition achieves high classification accuracy for waste, few studies have utilized image recognition for the classification of food waste.

In the process of handling food waste, lighting conditions are often less than ideal. This is because food waste sorting is conducted around the clock, with significant fluctuations in light levels. Even with the use of auxiliary lighting, it is difficult to ensure that lighting conditions remain within a reasonable range, which presents challenges for sorting. To overcome the limitations of single-modality sensors, recent research has shifted towards multimodal fusion strategies. Recent studies have demonstrated that integrating audio and visual information significantly enhances the precision of behavioral prediction models [17], while the fusion of multi-source sensor data can greatly improve model accuracy and stability in complex classification and estimation tasks [18]. For instance, Lu proposed a deep multimodal learning framework for municipal solid waste sorting, utilizing VGG16 for visual data and 1D CNNs for acoustic feature extraction [19]. Their results demonstrated that fusing deeply learned acoustic features with visual information significantly outperforms single-modality approaches.

Furthermore, regarding specific acoustic analysis for industrial machinery, sound-based monitoring has proven effective for characterising the state of complex machining processes. Similar to the waste crushing scenario, Li successfully applied multi-sensor feature extraction (including acoustic signals) to detect chatter phenomena in blade whirling milling [20]. Their study confirmed that frequency-domain features are highly effective in identifying the operational status of equipment under high-intensity material removal conditions. Therefore, by employing a multimodal logic fusion approach that combines image recognition with specific crusher audio analysis, the classification accuracy of kitchen waste is expected to improve significantly.

This paper proposes a method for identifying the texture types of food waste on the basis of independent intelligent recognition of images and audio, followed by logical fusion judgment. The specific research includes collecting food waste from restaurants in different regions during different seasons and conducting crushing tests on the waste. The image data are classified and identified via the MobileNetV3 + EMA model. Additionally, audio data from the crushing of food waste is collected, and a support vector machine (SVM) is employed as a classifier to categorize food waste on the basis of different textures. On the basis of the results from these two signal types, a logical fusion decision-making process is designed to increase the accuracy of food waste type identification.

## 2. Materials and Methods

### 2.1. Experimental Materials

**Figure 1:**
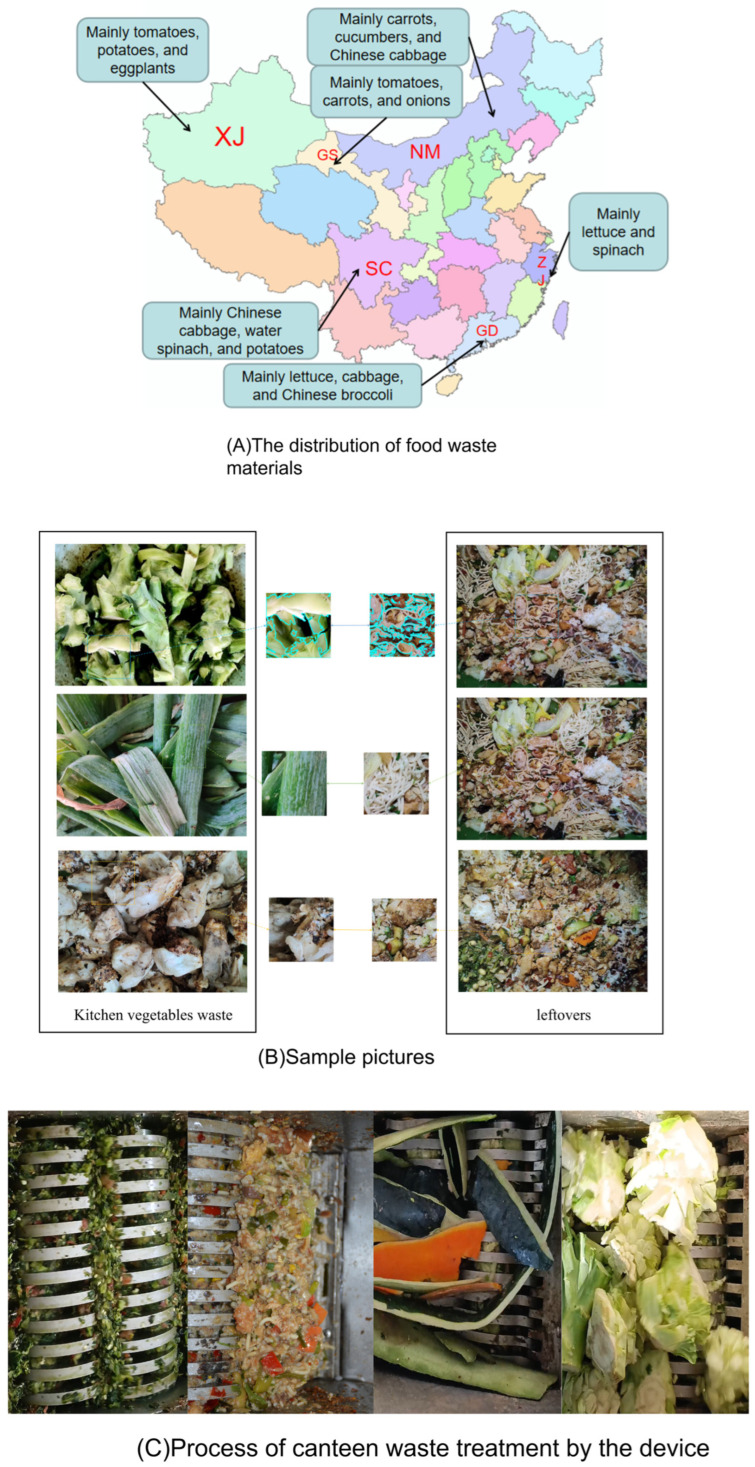
Collection and Disposal of Food Waste. (Red letters indicate the abbreviations of provinces).

The signal processing program is responsible for classifying and identifying waste through the analysis and learning of image and audio data. To ensure the accuracy of the program, it is important to collect a representative set of food waste images. These include images of kitchen waste and leftovers, as these are the most common types of waste in the food service industry. The dataset should also include many samples to cover various types of waste. To create a comprehensive learning sample, images of kitchen waste and leftovers generated by canteens during different seasons have been collected. As shown in Figure 1A, to ensure the applicability of the recognition model in different dietary habit regions, food waste materials are being collected from different regions during different seasons. The collected food waste and post-meal garbage samples are shown in Figure 1B. Figure 1C illustrates the process of managing food waste via this device.

### 2.2. Data Collection and Processing

#### 2.2.1. Image Acquisition and Processing

To ensure data quality and consistency, this paper used a vertically adjustable light source and standardized image acquisition equipment (MV-CA060-10GC, Hikvision, Hangzhou, China). However, in actual operation, image quality issues caused by human factors, such as improper focusing, overexposure, and shaking, still occurred. A manual review was subsequently conducted, and all problematic images were deleted. The final dataset consisted of 540 images, with 270 images depicting kitchen waste and another 270 images depicting leftover food. To expand the number of original images and increase sample diversity, this study used Python 3.10 (Python Software Foundation, Wilmington, DE, USA) to enhance the data. This was achieved by rotating and cropping the initial images, as well as adjusting the brightness range and contrast to increase the data sample size. After data augmentation, the image pixel dimensions were adjusted to a standardized size of 224 pixels × 224 pixels to facilitate model training. The final dataset contained 4027 sample images.

#### 2.2.2. Audio Capture and Processing

When audio data was collected, food waste was first sorted into kitchen waste and leftovers and then crushed using a crusher (SZ120, Shengda Light Industry Machinery Co., Ltd., Xinxiang, China).Audio recording devices (DS-2FP2020, Hikvision, Hangzhou, China) on the crusher were used to record the crushing sounds, which were then manually labeled as kitchen waste or leftovers. When audio processing began, libraries such as Librosa were used to read WAV-format audio files and convert them into digital signals.

Before applying the Fourier transform, the audio signal underwent preprocessing to improve signal quality and resolution. This included removing the DC component (subtracting the signal’s average value) and preemphasis (enhancing high-frequency components by weighting the signal). The audio signal was then divided into a series of short-time windows, each containing a stable signal. By applying a window function to each window, spectral leakage issues were reduced.

Subsequently, the Fourier transform was applied to each window to convert the time-domain signal into a frequency-domain signal [21,22]. This allowed the amplitude and phase information of the signal at different frequencies to be calculated. Next, based on the frequency-domain signal obtained from the Fourier transform, the energy spectrum of each window was calculated. This energy spectrum reflected the energy distribution within the frequency range and could be used to describe the spectral characteristics of the audio.

### 2.3. Image Feature Extraction

#### 2.3.1. ResNet18

ResNet18 is a deep convolutional neural network architecture consisting of 18 layers, including one convolutional layer, four residual blocks, one global average pooling layer, and one fully connected layer for classification.

#### 2.3.2. ECANET + ResNet18

Efficient channel attention (ECA) is a mechanism in convolutional neural networks designed to enhance the network’s ability to focus on important information. This is achieved by assigning weights to the channels within the network. The core of the ECA mechanism involves calculating the attention weights for each channel via global average pooling and a series of convolutional layers. This enables the network to effectively select the channels most relevant to a specific task while ignoring irrelevant information. ECA can be integrated into existing convolutional neural network architectures to improve model performance. In ResNet 18, the ECA module is added to the output of the final convolutional layer.

#### 2.3.3. SANET + ResNet18

The SA module is a structural variant of the attention mechanism designed to enhance the model’s ability to perceive features. By integrating channel attention and spatial attention, the model can capture important information in images more comprehensively. It simultaneously considers the relationships between channels and spatial relationships, thereby improving the model’s perceptual capabilities. Similar to the SE module, the SA module is placed after the residual block.

#### 2.3.4. MobileNetV3

MobileNetV3 is a lightweight convolutional neural network architecture optimized via Network Architecture Search (NAS). It consists of a stem convolutional layer, a stack of inverted residual blocks with linear bottlenecks and Squeeze-and-Excitation (SE) modules, a global average pooling layer, and a final fully connected layer for classification.

#### 2.3.5. MobileNetV3 + EMA

To further enhance the model’s feature extraction capabilities within complex industrial environments, we introduce the Efficient Multi-Scale Attention (EMA) mechanism into the MobileNetV3-Small architecture. The EMA module is designed to overcome the high computational costs associated with traditional attention mechanisms while preserving the ability to capture long-range dependencies.

The EMA module primarily aggregates features through grouped convolution and cross-spatial learning. First, the input feature map is grouped along the channel dimension (Factor = 8) to reduce computational complexity. The module then splits into two parallel branches: one branch utilizes 1 × 1 and 3 × 3 convolutions to encode global spatial information, while the other employs adaptive average pooling in horizontal and vertical directions to capture pixel-level spatial dependencies. Finally, a Cross-Spatial Information Aggregation method fuses the outputs of these two branches via matrix multiplication to generate the final attention weight map.

The detailed structure of the proposed MobileNetV3 + EMA architecture is presented in Table 1. In the proposed MobileNetV3 + EMA architecture, the EMA module is embedded immediately after the backbone’s feature extraction layers and before the global average pooling layer. Specifically, once the MobileNetV3 completes feature extraction through its stack of 11 Inverted Residual Blocks (outputting a 576 × 7 × 7 feature map), the data enters the EMA module for feature recalibration before proceeding to classification. This design enables the network to focus on feature channels containing critical food waste texture information without significantly increasing the number of parameters.

#### 2.3.6. Hyperparameter Design

To ensure the reliability and universality of the model, this paper divided the sample set into a test set and a training set. The test set included 552 randomly selected samples, accounting for 20% of the total number of samples, while the remaining 3475 samples were used for training. This partitioning effectively evaluates the model’s performance on new data and helps avoid overfitting the training set.

First, the model’s performance was optimized through careful selection of hyperparameters. Based on the experimental requirements, the Stochastic Gradient Descent (SGD) optimizer was employed with a momentum parameter of 0.9, which facilitated faster convergence and reduced oscillation during training. An initial learning rate of 0.005 was selected to ensure stable weight updates. To further prevent overfitting and enhance the model’s fine-tuning capability in later stages, a stepwise learning rate decay strategy was implemented, where the learning rate was reduced by a factor of 0.1 every 15 epochs. Additionally, a batch size of 32 was chosen to balance memory efficiency with gradient estimation accuracy. The model was trained for a total of 50 epochs to ensure complete convergence.

The experimental environment was configured to maximize computational efficiency. To accelerate the training of the deep neural network and handle the extensive matrix operations required by the MobileNetV3 architecture and the EMA module, a high-performance GPU was utilized as the execution environment. This setup significantly utilized parallel computing capabilities to speed up the parameter update process.

### 2.4. Audio Feature Extraction

Audio feature extraction uses an SVM (support vector machine), which is a powerful classification algorithm. Its basic idea is to map the dataset to a high-dimensional space, find the optimal hyperplane, and divide the data into two categories. This hyperplane is composed of many support vectors, which are the closest data points. For the classification stage, a Support Vector Machine (SVM) with a Radial Basis Function (RBF) kernel was employed. The hyperparameters were optimized using grid search, resulting in a penalty parameter C of 1.0 and a kernel coefficient Gamma of ‘scale’. Prior to classification, all extracted frequency domain features underwent Z-score normalization to eliminate dimensional differences and ensure model convergence.

### 2.5. Model Evaluation Indicators

Model evaluation metrics are used to assess the performance and effectiveness of classification models and deep learning models. The classification models were evaluated by employing four metrics, including accuracy, F1 score, recall, and precision [23,24,25].

Accuracy (Acc): As one of the most widely used evaluation metrics in classification problems, Formula (1) represents the proportion of correctly predicted samples by the classifier out of the total number of samples.

(1) $$\begin{array}{c} Acc=\frac{TP+TN}{TP\;+\;FP+FN+TN} \end{array}$$

Precision (*P*): Formula (2) is used to measure the accuracy of positive prediction results.

(2) $$\begin{array}{c} P=\frac{TP}{TP+FP} \end{array}$$

Recall (*R*) Formula (3) is used to measure the proportion of correctly predicted positive samples.

(3) $$\begin{array}{c} R=\frac{TP}{TP+FN} \end{array}$$

F1 score (F1): Formula (4) is an indicator used to measure the overall accuracy and recall, which is their harmonic mean.

(4) $$\begin{array}{c} F1=\frac{2PR}{P+R} \end{array}$$

In these formulas, TP (true positive) represents the number of correctly predicted positive samples, TN (true negative) represents the number of correctly predicted negative samples, FP (false positive) represents the number of incorrectly predicted positive samples, and FN (false negative) represents the number of incorrectly predicted negative samples.

### 2.6. Multimodal Logic Fusion Method

**Figure 2:**
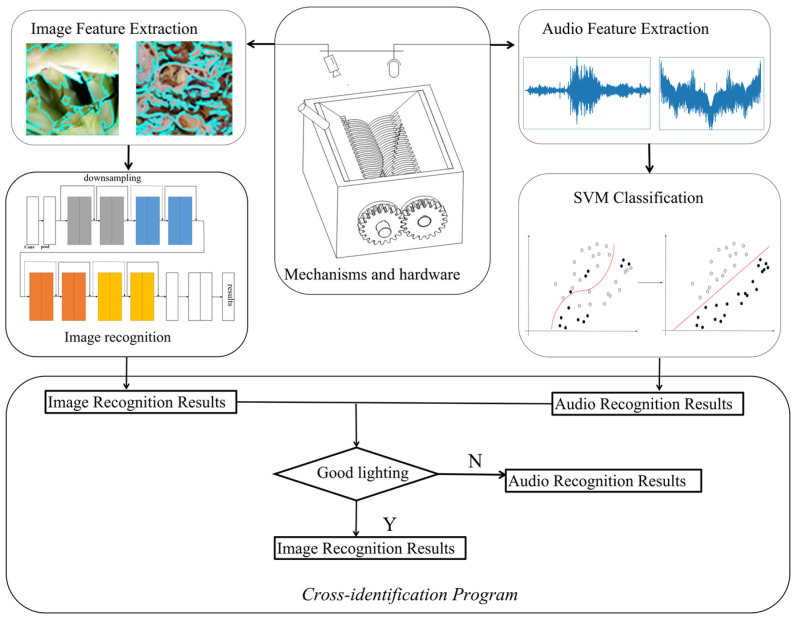
Framework diagram of the Canteen waste identification system. (The arrows indicate the direction of data flow and the processing steps of the system).

For food waste, certain materials can cause semantic confusion and contradictions between image recognition results and audio recognition results. This phenomenon indicates that directly fusing image and audio data as input parameters for learning may actually reduce accuracy. To address this issue, this paper employs a multimodal logical fusion method to distinguish the texture of food waste, as illustrated in Figure 2.

The multimodal logical fusion method specifically involves selecting an appropriate recognition approach on the basis of real-time environmental lighting intensity. Since excessively weak or strong lighting can cause fluctuations in image recognition rates, combining image recognition with audio recognition is essential. Given that audio recognition rates remain relatively stable and are not significantly affected by variations in lighting, the image recognition result is prioritized when lighting is within the optimal range; otherwise, the audio recognition result is adopted as the final decision.

### 2.7. Experimental Platform

The experiments were implemented using the PyTorch deep learning framework on a high-performance local workstation. The hardware configuration featured an NVIDIA GeForce RTX 4060 GPU (NVIDIA, Santa Clara, CA, USA) with 8 GB of video memory, utilizing CUDA 11.8 for hardware acceleration. The operating system was Windows, and the software environment relied on Python 3.10 (Python Software Foundation, Wilmington, DE, USA) and PyTorch 2.0.0 (Meta AI, Menlo Park, CA, USA) for model construction and evaluation.

## 3. Results

### 3.1. Figures, Tables and Schemes

**Figure 3:**
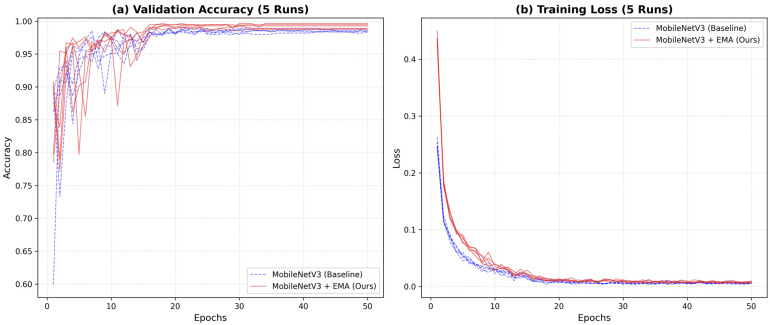
Comparison of accuracy and loss rate.

To identify the optimal architecture for industrial food waste sorting, this paper conducts a comprehensive comparison of five network models. These include the classic ResNet18 baseline and its attention-enhanced variants (ECANET + ResNet18, SANET + ResNet18) [10,26,27,28], alongside the state-of-the-art lightweight MobileNetV3-Small and our proposed MobileNetV3 + EMA. All models were trained and tested under the exact same hardware environment (NVIDIA GeForce RTX 4060). To ensure statistical reliability, the reported results represent the average of five independent training runs. The overall quantitative comparison is summarized in Table 2, while the specific training dynamics of the lightweight models are visualized in Figure 3.

As detailed in Table 2, the proposed MobileNetV3 architectures significantly outperform the ResNet-based variants in both precision and convergence. While the ResNet family achieves respectable accuracy (peaking at 97.28%), their training loss remains relatively high, ranging from 3.76% to 4.35%. In contrast, the MobileNetV3 + EMA achieves the highest average accuracy of 99.46% (±0.18%) and a remarkably low loss of 0.82%. This drastic reduction in loss—nearly 5 times lower than the ResNet baselines—indicates that the lightweight model fits the food waste data distribution with much higher confidence and precision. To further analyze the convergence behavior, Figure 3 illustrates the training curves of the baseline MobileNetV3-Small (blue dashed lines) versus the proposed MobileNetV3 + EMA (red solid lines) over 50 epochs. As observed in Figure 3a, the EMA mechanism improves stability, enabling the model to consistently reach higher validation accuracy in the later stages. Similarly, the loss curves in Figure 3b demonstrate that the proposed model exhibits a convergence trajectory and loss magnitude highly comparable to the baseline. This confirms that the integration of the multi-scale attention mechanism facilitates feature learning while maintaining the robust training stability of the original lightweight architecture.

From an industrial deployment perspective, the trade-off between model size and speed is critical. As shown in Table 2, the ResNet family exhibits a heavy computational footprint, with model sizes exceeding 44 MB and parameter counts over 11 M. In stark contrast, the MobileNetV3 + EMA drastically reduces the model size to 6.41 MB, representing a reduction of approximately 85% in storage requirements. Although ResNet18 achieves the highest raw inference speed (357 FPS) due to its high parallelism on desktop GPUs, the proposed model maintains a robust speed of 180 FPS. This is well above the real-time requirement (typically 25–30 FPS) for conveyor belt monitoring. Consequently, the MobileNetV3 + EMA is selected as the backbone for the proposed system, offering the optimal balance of accuracy, compact size, and real-time performance.

### 3.2. Imaging Results and Analysis

Illuminance can be measured via an illuminance meter, which detects the intensity of light. Typically, the indoor illuminance during the day is approximately 500 lux. As the illuminance increases, the average brightness also increases. To calculate the average brightness of a color image, it can be converted to a grayscale image, after which the average brightness of the grayscale image can be computed. In this study, an illuminance meter was utilized to assess the shooting environment, and a shredder was employed to process food waste, with the entire procedure recorded on video. To construct the Logic Validation Set, we collected 143 original images. To enhance sample diversity, each original image was cropped into 25 sub-images, resulting in a total of 3575 sub-images with standardized dimensions of 224 × 224 pixels. These images were subsequently identified and verified by the trained MobileNetV3 + EMA network.

**Figure 4:**
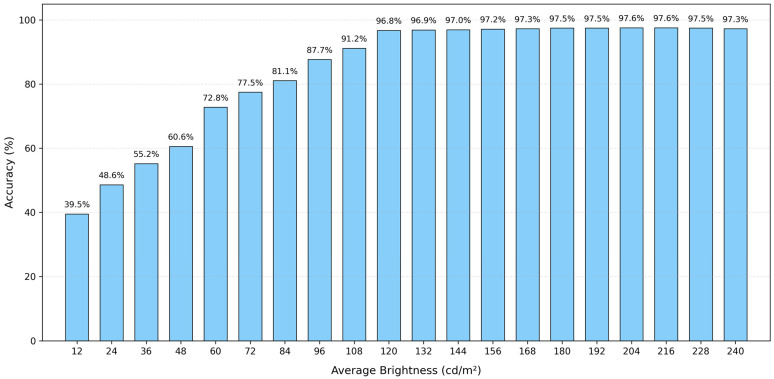
Accuracy of model verification under different average brightness values.

Under the same lighting conditions, the average brightness of the original image is approximately 120 cd m^−2^. To simulate various environmental lighting conditions, the average brightness of the original image was adjusted while keeping other image parameters constant, resulting in the creation of 20 test sets corresponding to different brightness levels. The MobileNetV3 + EMA network model was employed for recognition, and the results are presented in Figure 4. When the average brightness was set between 120 cd m^−2^ and 240 cd m^−2^, the accuracy consistently exceeded 96%, peaking at 97.6%. This indicates that the MobileNetV3 + EMA model demonstrated exceptional stability and robustness under sufficient lighting conditions. However, when the brightness falls below 84 cd m^−2^, the model’s recognition accuracy decreases to less than 80%, rendering it unable to identify and classify food waste accurately.

This is because kitchen waste typically includes fruit peels, stems, and leaves. These components are primarily food byproducts, characterized by their varying colors and shapes, and they tend to have relatively large volumes, as illustrated in Figure 1B. In terms of shape, kitchen waste is generally composed of scattered fragments and fibres, whereas leftovers lack a fixed shape and typically consist of a mixture of solids and liquids, as depicted in Figure 1B. Under extreme lighting conditions, such as intense brightness, the fragments in kitchen waste may become distorted or lose essential details and features, making it challenging for the model to accurately extract and identify object characteristics.

### 3.3. Audio Results and Analysis

**Figure 5:**
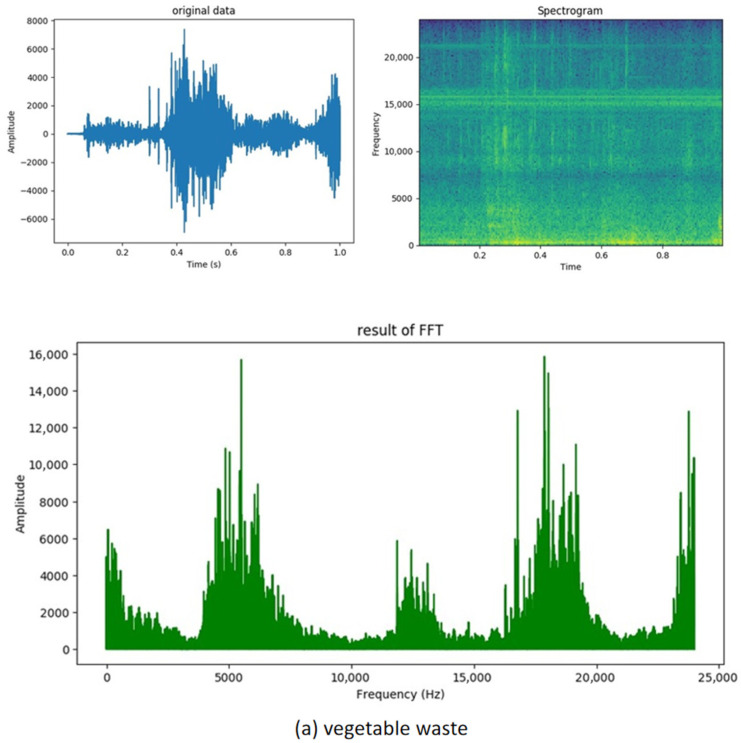

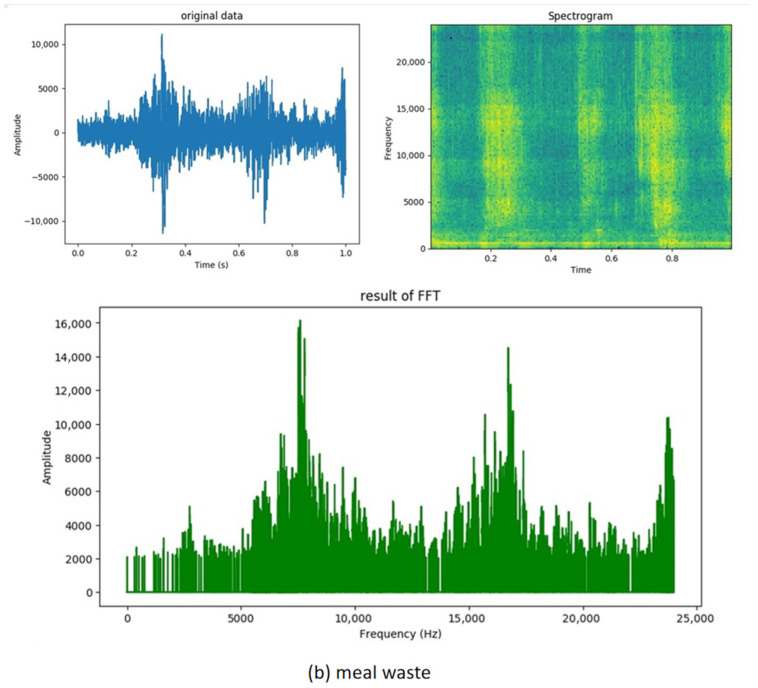
Audio waveform, spectrum, and FFT results for cafeteria garbage.

As illustrated in Figure 5, converting the audio signal into a spectrogram allows for the extraction of the energy distributions of different frequencies within each time window, thereby effectively capturing the frequency characteristics of the audio. The audio data are segmented into 1-s intervals, corresponding to the one-second rotation cycle of the crusher, which ensures the integrity of the audio. The kitchen waste dataset comprises 2660 samples, whereas the leftover dataset contains 2600 samples. During the data segmentation process, it is essential to label each sample with its corresponding category to facilitate subsequent classification tasks. To mitigate overfitting and enhance data diversity, the order of the data should be randomly shuffled. This approach helps the model better learn the features of the audio data and reduces bias during training. A random sample of 720 audio files is extracted from the dataset to serve as the validation set, while the remaining files are utilized as the training set. This methodology enables the model to be evaluated and tested for its ability to generalize to new data.

The experimental results demonstrate that the audio model provides a reliable fallback mechanism. Beyond the overall Accuracy of 80%, we specifically evaluated the model’s stability using Precision (0.82), Recall (0.78), and the F1-score (0.80). Unlike accuracy, which can be misleading if the model is biased toward a majority class, the high F1-score (0.80) confirms a strong balance between precision and recall, indicating that the model effectively learns features for both waste categories without significant bias. Specifically, the recognition rates for ‘leftovers’ (78%) and ‘kitchen waste’ (82%) are closely aligned. This class-wise consistency ensures that the audio modality remains robust and trustworthy for all waste types, particularly when the visual model fails due to extreme lighting conditions.

There are 359 audio frequencies in the leftover category, of which 280 were correctly identified, resulting in an identification rate of 78%. In the kitchen waste category, there are 361 audio frequencies, with 296 correctly identified, yielding an identification rate of 82%. This misidentification can be attributed to the significant variation in the color and texture of kitchen waste, which includes soft, hard, and sticky components. As shown in Figure 1B, the images in the leftover category exhibit obvious oil stains, indicating a relatively high oil content. Consequently, when the shredder processes the softer parts of kitchen waste, the audio frequencies tend to be more similar to those of the leftover category. Overall, the model demonstrates strong performance in classifying kitchen waste and leftover categories, achieving high recognition rates. Thus, the model exhibits high accuracy and reliability in audio classification tasks.

### 3.4. Results of Multimodal Logic Fusion Judgment

**Figure 6:**
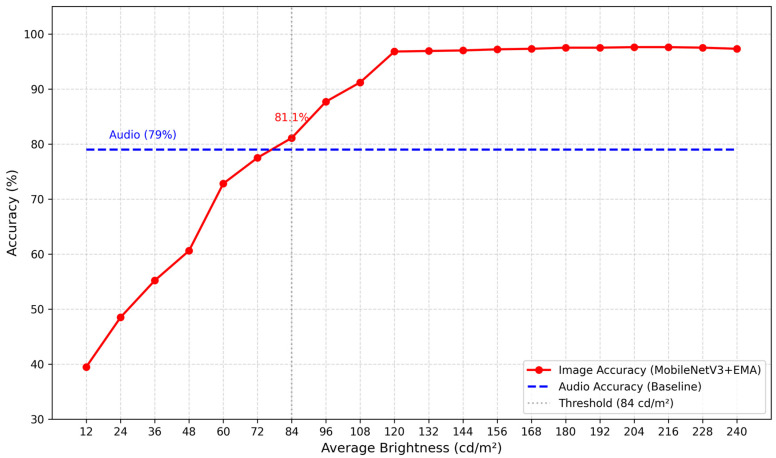
Verification accuracy of the coupling model under different average brightness levels.

The audio and images obtained in Section 3.2 are separated, matched, and annotated. The fragmented audio is extracted while keeping the images unchanged. Subsequently, 143 fragmented audio clips were extracted and identified, achieving a final accuracy of 79%. As illustrated in Figure 6, when the average brightness of the images exceeds 84 cd m^−2^, the accuracy consistently surpasses 79%. At this stage, the accuracy of image recognition exceeds that of audio recognition, allowing for the direct output of image recognition results. However, when the average brightness falls below 84 cd m^−2^, the accuracy of image recognition decreases relative to that of audio recognition, and the audio recognition results are adopted. Notably, at an average brightness of 12 cd m^−2^, the accuracy improvement reaches 39.5%.

### 3.5. Logical Verification

**Figure 7:**
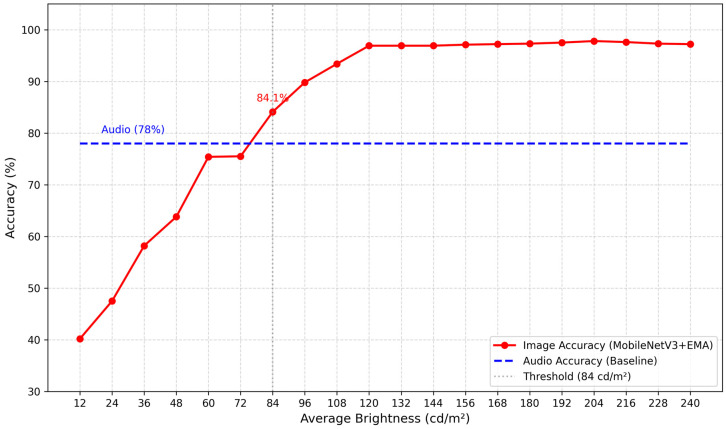
Test set data.

The process and quantity of image dataset production in the independent test set are the same as those in Section 3.2. The process and quantity of audio data production are the same as those in Section 3.3. The fusion logic proposed in Section 3.4 is verified under the independent test set, and the results are shown in Figure 7. It can be seen from the figure that in the independent test set, the accuracy rate is 78% when only audio is used, 88.48% when only images are used, and 90.25% when the fusion logic proposed in 3.4 is used. Thus, it can be seen that this fusion logic is effective.

## 4. Conclusions

This paper proposes a multimodal logic fusion method for texture discrimination in food waste processing. The proposed method first learns from various types of data sources, establishes the priority of discrimination criteria in areas where each data source excels, and ultimately employs logical settings to distinguish the texture of food waste. This approach effectively addresses the semantic ambiguity that arises when different data sources describe the same object.

In terms of image recognition, the performances of five distinct models—including ResNet18, its variants, MobileNetV3, and MobileNetV3 + EMA—were compared. The results demonstrated that the MobileNetV3 + EMA model achieved the highest accuracy of 99.46%, with improvements of 2.73% and 0.66% over the ResNet18 baseline and standard MobileNetV3, respectively, and exhibited the lowest loss value of 0.82%. Furthermore, this model maintains robust recognition performance while being compact in size (6.41 MB) and efficient (180 FPS).

For audio recognition, the model utilizes fast Fourier transform (FFT) feature extraction combined with SVM classification methods. The results indicate that the model achieves high accuracy in classifying broken food waste, with an accuracy rate of 0.80, a recall rate of 0.78, and an F1 score of 0.80. Notably, when the average brightness is below 84 cd m^−2^, the model’s accuracy surpasses that of the original image recognition model. Specifically, at an average brightness of 12 cd m^−2^, the accuracy improved by 39.5%. Within the brightness range of 120 cd m^−2^ to 240 cd m^−2^, the accuracy rates consistently exceeded 96%.

Limitations and Future Work While the proposed multimodal fusion method demonstrates high accuracy and robustness, several limitations remain. First, the current system is designed for a binary classification task (kitchen waste vs. leftovers) using a specific type of industrial crusher. The acoustic features may vary significantly with different hardware configurations or waste categories. Second, although the logic fusion strategy is generalizable, the specific brightness threshold (84 cd m^−2^) is environment-dependent and may require recalibration for different factory lighting setups. Future work will focus on expanding the dataset to include multi-category waste and investigating transfer learning techniques to adapt the model efficiently to different crushing equipment and broader industrial noise environments.

## Figures and Tables

**Table 1:** MobileNetV3 + EMA model structure table.

Layer Name	Input Dimension	Output Dimension	Operation Description
Input	3 × 224 × 224	3 × 224 × 224	Preprocessed RGB image input.
Features (Stem)	3 × 224 × 224	16 × 112 × 112	First Conv3 × 3, stride 2, BN, h-swish.
Bottleneck 1–11	16 × 112 × 112	576 × 7 × 7	Stack of 11 Inverted Residual Blocks (SE modules embedded in specific blocks).
Conv_last	96 × 7 × 7	576 × 7 × 7	Pointwise Conv1 × 1 to expand channels, BN, h-swish.
EMA Module	576 × 7 × 7	576 × 7 × 7	Efficient Multi-Scale Attention: 1. Grouping (factor = 8); 2. Parallel 1 × 1 & 3 × 3 Convs; 3. Coordinate Pooling (H/W); 4. Cross-spatial Aggregation & Sigmoid re-weighting.
AvgPool	576 × 7 × 7	576 × 1 × 1	Adaptive Average Pooling to 1 × 1.
Flatten	576 × 1 × 1	576	Flatten feature map to vector.
Classifier (Head)	576	2	Sequential: 1. Linear (576 -> 1024), h-swish; 2. Dropout (*p* = 0.2); 3. Linear (1024 -> 2) for final classification.

**Table 2:** Model Comparison.

Model	Accuracy (%)	Loss (%)	Params (M)	Model Size (MB)	Speed (FPS)
ResNet18	96.73	4.35	11.18	44.79	357
ECANET + ResNet18	97.28	3.76	11.18	44.79	249
SANET + ResNet18	96.92	4.17	11.27	45.15	190
MobileNetV3-Small	98.80	0.59	1.52	6.20	190
MobileNetV3 + EMA (Ours)	99.46	0.82	1.57	6.41	180

## Data Availability

Data is contained within the article or Supplementary Materials.

## Supplementary Materials

The following supporting information can be downloaded at: https://www.mdpi.com/article/10.3390/s26030851/s1, Figure S1: Process of canteen waste treatment by the device; Figure S2: Sample pictures; Figure S3: Original sub-images used for the system flowchart.
