# Supplementary material for: Utilizing Multimodal Logic Fusion to Identify the Types of Food Waste Sources
Source: Sensors (Basel). 2026 Jan 28;26(3):851. doi: 10.3390/s26030851 (PMC12899810; doi:10.3390/s26030851)
Supplement: Supplementary file 1 [file sensors-26-00851-s001.zip › sensors-4065759-supplementary.pdf]

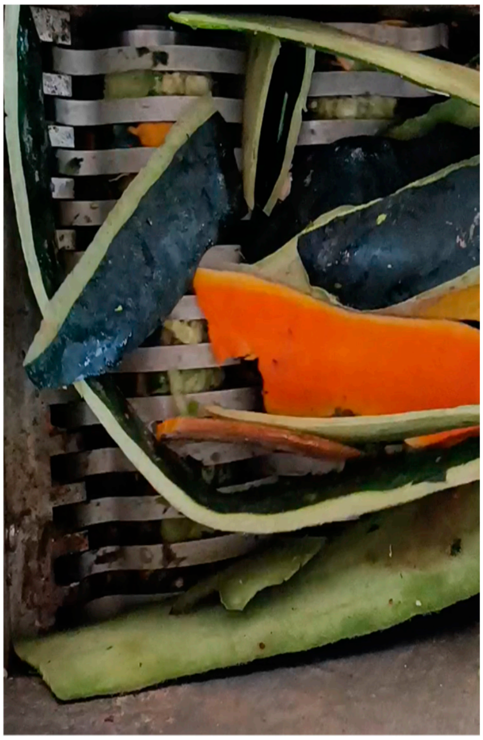

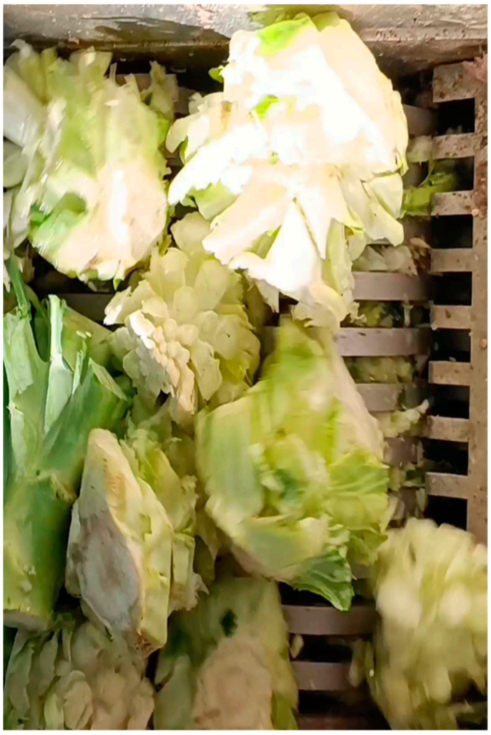

Figure S1（a） Figure S1（b）

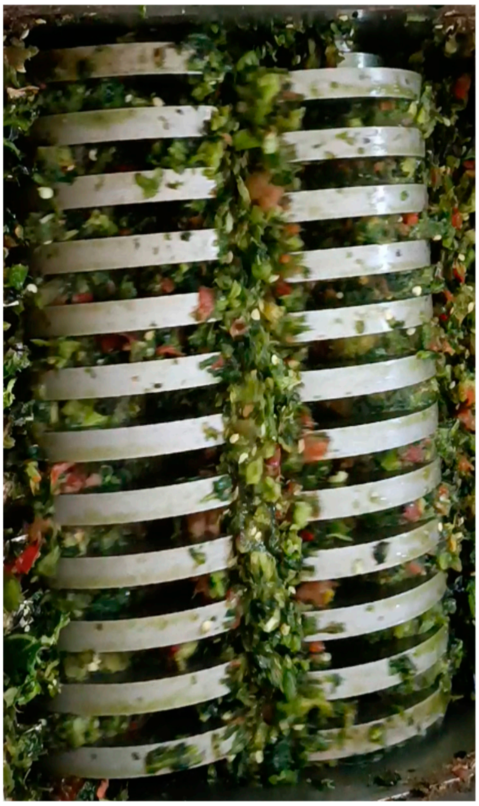

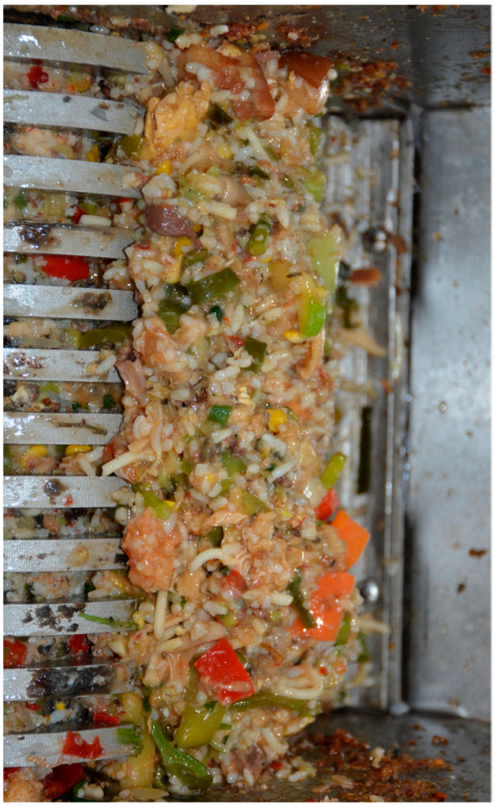

Figure S1（c） Figure S1（d）

Figure S1. Process of canteen waste treatment by the device

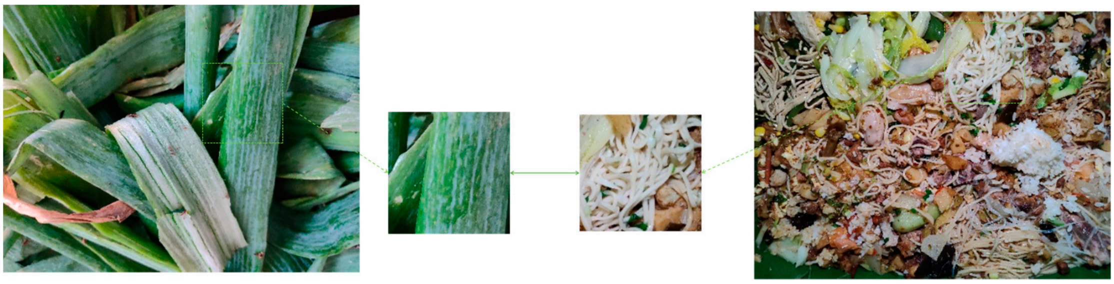

Figure S2.（a）

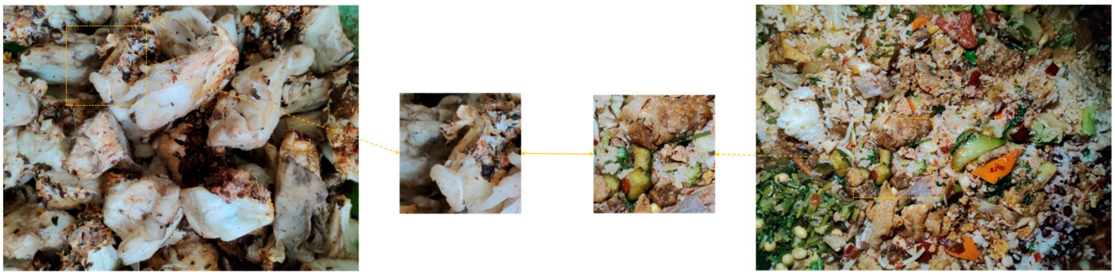

Figure S2.（b）

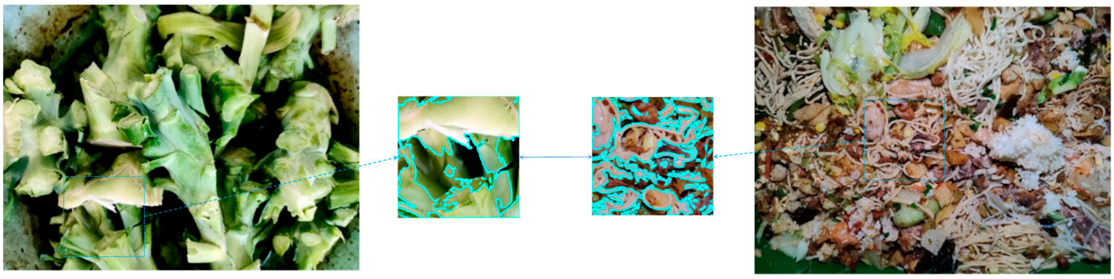

Figure S2.（c）

Figure S2. Sample pictures

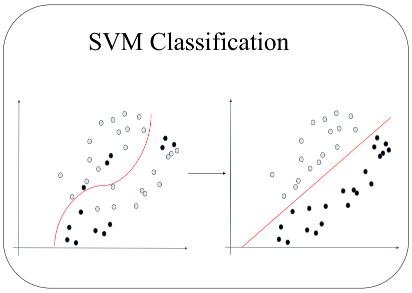

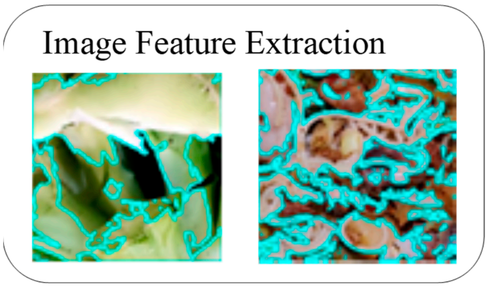

Figure S3（a）

Figure S3（b）

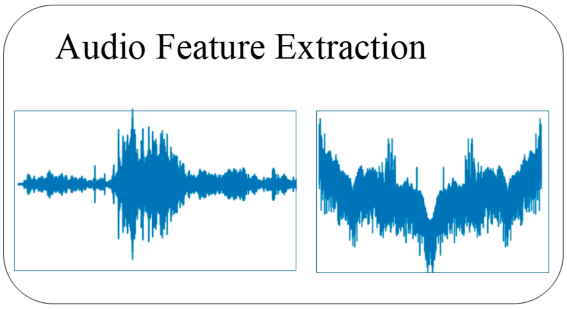

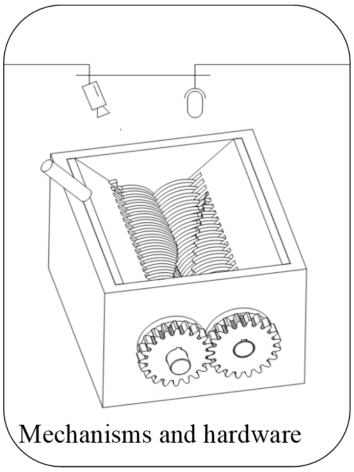

Figure S3（c）

Figure S3（d）

Figure S3. Original sub-images used for the system flowchart
